# Supplementary material for: Factors associated with hypertensive disorders of pregnancy in sub-Saharan Africa: A systematic and meta-analysis
Source: PLoS One. 2020 Aug 19;15(8):e0237476. doi: 10.1371/journal.pone.0237476 (PMC7437911; doi:10.1371/journal.pone.0237476)
Supplement: S1 Table — (DOCX) [file pone.0237476.s002.docx]

| **Table S1: Searching terms for research articles** | |
| --- | --- |
| **Databases** | **Searching terms** |
| **Medline** | hypertension, pregnancy-induced/ or eclampsia/ or hellp syndrome/ or pre-eclampsia/ AND eclamp*.mp. AND (preeclamp* or pre-eclamp*).mp. AND (hypertens* adj3 (preg* or gestat*)).mp. OR "africa south of the sahara"/ or africa, eastern/ or burundi/ or eritrea/ or ethiopia/ or kenya/ or rwanda/ or somalia/ or south sudan/ or sudan/ or tanzania/ or uganda/ (Subsahara* or Sub-Sahara* or Ethiopia* or Kenya* or Somalia* or Sudan* or Tanzania* or Uganda* or Eriteria* or Burundi* or Angola* or Benin* or Botswana* or Burkina Faso* or Cameroon* or Cape Verde* or Central African Republic* or Chad* or Comoros* or Congo * or Cote d'Ivoire* or Djibouti* or Equatorial Guinea* or Gabon* or Gambia * or Ghana* or Guinea * or Lesotho* or Liberia * or Madagascar* or Malawi * or Mali* or Mauritania* or Mauritius* or Mozambique* or Namibia * or Niger* or Nigeria* or Rwanda* or Sao Tome or Principe* or Senegal* or Seychelles* or Sierra Leone* or Somalia* or South Africa* or Sudan* or Swaziland * or Tanzania* or Togo * or Uganda* or Western Sahara Zambia * or Zimbabwe*).mp. AND limit to (english language and yr="2000 -Current") |
| **Maternity and infant care** | hypertension, pregnancy-induced.mp. [mp=abstract, heading word, title] OR eclampsia.mp. [mp=abstract, heading word, title] OR hellp syndrome.mp. [mp=abstract, heading word, title] OR pre-eclampsia.mp. [mp=abstract, heading word, title] OR eclamp*.mp. OR (preeclamp* or pre-eclamp*).mp. OR (hypertens* adj3 (preg* or gestat*)).mp. AND (Subsahara* or Sub-Sahara* or Ethiopia* or Kenya* or Somalia* or Sudan* or Tanzania* or Uganda* or Eriteria* or Burundi* or Angola* or Benin* or Botswana* or Burkina Faso* or Cameroon* or Cape Verde* or Central African Republic* or Chad* or Comoros* or Congo * or Cote d'Ivoire* or Djibouti* or Equatorial Guinea* or Gabon* or Gambia * or Ghana* or Guinea * or Lesotho* or Liberia * or Madagascar* or Malawi * or Mali* or Mauritania* or Mauritius* or Mozambique* or Namibia * or Niger* or Nigeria* or Rwanda* or Sao Tome or Principe* or Senegal* or Seychelles* or Sierra Leone* or Somalia* or South Africa* or Sudan* or Swaziland * or Tanzania* or Togo * or Uganda* or Western Sahara Zambia * or Zimbabwe*).mp. AND limit to (english language and yr="2000 -Current") |
| **CINAHL Complete** | (preg* or gestat*) OR (hypertens* OR (preeclamp* or pre-eclamp*) OR eclamp* OR hypertension, pregnancy-induced or eclampsia or hellp syndrome or pre-eclampsia AND (Subsahara* or Sub-Sahara* or Ethiopia* or Kenya* or Somalia* or Sudan* or Tanzania* or Uganda* or Eriteria* or Burundi* or Angola* or Benin* or Botswana* or Burkina Faso* or Cameroon* or Cape Verde* or Central African Republic* or Chad* or Comoros* or Congo * or Cote d'Ivoire* or Djibouti* or Equatorial Guinea* or Gabon* or Gambia * or Ghana* or Guinea * or Lesotho* or Liberia * or Madagascar* or Malawi * or Mali* or Mauritania* or Mauritius* or Mozambique* or Namibia * or Niger* or Nigeria* or Rwanda* or Sao Tome or Principe* or Senegal* or Seychelles* or Sierra Leone* or Somalia* or South Africa* or Sudan* or Swaziland * or Tanzania* or Togo * or Uganda* or Western Sahara Zambia * or Zimbabwe*).mp. AND limit to (english language and yr="2000 -Current") |
| **EMBASE** | hypertension, pregnancy-induced.mp. or maternal hypertension/ OR eclampsia/HELLP syndrome/ OR  pre-eclampsia.mp. or preeclampsia/OR eclamp*.mp. OR (preeclamp* or pre-eclamp*).mp. OR (hypertens* adj3 (preg* or gestat*)).mp. AND "africa south of the sahara"/ or africa, eastern/ or burundi/ or eritrea/ or ethiopia/ or kenya/ or rwanda/ or somalia/ or south sudan/ or sudan/ or tanzania/ or uganda/  (Subsahara* or Sub-Sahara* or Ethiopia* or Kenya* or Somalia* or Sudan* or Tanzania* or Uganda* or Eriteria* or Burundi* or Angola* or Benin* or Botswana* or Burkina Faso* or Cameroon* or Cape Verde* or Central African Republic* or Chad* or Comoros* or Congo * or Cote d'Ivoire* or Djibouti* or Equatorial Guinea* or Gabon* or Gambia * or Ghana* or Guinea * or Lesotho* or Liberia * or Madagascar* or Malawi * or Mali* or Mauritania* or Mauritius* or Mozambique* or Namibia * or Niger* or Nigeria* or Rwanda* or Sao Tome or Principe* or Senegal* or Seychelles* or Sierra Leone* or Somalia* or South Africa* or Sudan* or Swaziland * or Tanzania* or Togo * or Uganda* or Western Sahara Zambia * or Zimbabwe*).mp. AND limit 12 to (english language and yr="2000 -Current") |
