# Supplementary material for: Factors associated with hypertensive disorders of pregnancy in sub-Saharan Africa: A systematic and meta-analysis
Source: PLoS One. 2020 Aug 19;15(8):e0237476. doi: 10.1371/journal.pone.0237476 (PMC7437911; doi:10.1371/journal.pone.0237476)
Supplement: S2 Table — (DOCX) [file pone.0237476.s003.docx]

| **Table S2 : Quality Assessment for research article** | | | | | | | | | | | | | |
| --- | --- | --- | --- | --- | --- | --- | --- | --- | --- | --- | --- | --- | --- |
| **S.No** | **Authors** | **Question 1** | **Question 2** | **Question 3** | **Question 4** | **Question 5** | **Question 6** | **Question 7** | **Question 8** | **Question 9** | **Question 10** | **Total(10)** | **Quality of paper** |
| **1** | **Jennifer Murray/2018** | **Yes** | **Yes** | **Yes** | **Yes** | **Yes** | **Yes** | **Yes** | **Yes** | **Yes** | **Clear** | **8** | **High** |
| **2** | **Tarkie Abebe Walle/2019** | **Yes** | **Yes** | **Yes** | **Yes** | **Yes** | **Yes** | **Yes** | **Yes** | **Yes** | **Clear** | **8** | **High** |
| **3** | **Swati Singh/2014** | **Yes** | **Yes** | **Yes** | **Yes** | **Yes** | **Yes** | **Yes** | **Yes** | **Yes** | **Clear** | **10** | **High** |
| **4** | **Hadiza A. Agbo/2016** | **Yes** | **No** | **Yes** | **Yes** | **Yes** | **Yes** | **Yes** | **Yes** | **Yes** | **unclear** | **7** | **Medium** |
| **5** | **Wubanchi Terefe/2015** | **Yes** | **Yes** | **Yes** | **Yes** | **Yes** | **Yes** | **Yes** | **Yes** | **Yes** | **Clear** | **8** | **High** |
| **6** | **C. T. Ndao/2009** | **Yes** | **No** | **Yes** | **Yes** | **Yes** | **Yes** | **Yes** | **Yes** | **Yes** | **Clear** | **9** | **High** |
| **7** | **Tesfaye Abera Gudeta/2018** | **Yes** | **Yes** | **Yes** | **Yes** | **Yes** | **Yes** | **Yes** | **Yes** | **Yes** | **unclear** | **8** | **Medium** |
| **8** | **Pierre Marie Tebeu/2011** | **Yes** | **Yes** | **Yes** | **Yes** | **Yes** | **Yes** | **Yes** | **Yes** | **Yes** | **Clear** | **8** | **Medium** |
| **9** | **Hailemariam Berhe Kahsay/2018** | **Yes** | **Yes** | **Yes** | **Yes** | **Yes** | **Yes** | **Yes** | **Yes** | **Yes** | **Clear** | **10** | **High** |
| **10** | **Deborah van Middendorp/2013** | **Yes** | **Yes** | **Yes** | **Yes** | **Yes** | **Yes** | **Yes** | **Yes** | **Yes** | **Clear** | **8** | **Medium** |
| **11** | **A. A. Ali/2014** | **Yes** | **Yes** | **Yes** | **Yes** | **Yes** | **Yes** | **Yes** | **Yes** | **Yes** | **Clear** | **9** | **High** |
| **12** | **Akwilina W. Mwanri/2015** | **Yes** | **Yes** | **Yes** | **Yes** | **Yes** | **Yes** | **Yes** | **Yes** | **Yes** | **Clear** | **8** | **High** |
| **13** | Getinet Ayele et al. (2016) | **Yes** | **Yes** | **Yes** | **Yes** | **Yes** | **Yes** | **Yes** | **Yes** | **Yes** | **Clear** | **9** | **High** |
| **14** | Mastewal Arefaynie Temesgen (2017) | **Yes** | **Yes** | **Yes** | **Yes** | **Yes** | **Yes** | **Yes** | **Yes** | **Yes** | **Unclear** | **8** | **Moderate** |
| **15** | Emmanuel Ratemo Omenya et al. (2018) | **Yes** | **Yes** | **Yes** | **Yes** | **Yes** | **Yes** | **No** | **Yes** | **Yes** | **Unclear** | **8** | **Moderate** |
| **16** | Samuel Azubuike and Ibrahim Danjuma (2017) | **Yes** | **Yes** | **Yes** | **No** | **Yes** | **Yes** | **Yes** | **Yes** | **Yes** | **Unclear** | **7** | **Moderate** |
| **17** | P.N Ebeigbe et al (2007) | **Yes** | **Yes** | **Yes** | **Yes** | **Yes** | **Yes** | **Yes** | **Yes** | **Yes** | **Clear** | **8** | **High** |
| **18** | Zenebe Wolde et al. (2011) | **Yes** | **Yes** | **Yes** | **Yes** | **Yes** | **Yes** | **Yes** | **Yes** | **Yes** | **Clear** | **9** | **High** |
| **19** | Liyew Mekonnen et al. (2018) | **Yes** | **Yes** | **Yes** | **Yes** | **Yes** | **Yes** | **Yes** | **Yes** | **Yes** | **Clear** | **9** | **High** |
| **20** | W.K.B.A. Owiredu et al. (2012) | **Yes** | **Yes** | **Yes** | **Yes** | **Yes** | **Yes** | **Yes** | **Yes** | **Yes** | **Unclear** | **8** | **Moderate** |
| **21** | Leta Hinkosa et al. (2020) | **Yes** | **Yes** | **Yes** | **Yes** | **Yes** | **Yes** | **Yes** | **Yes** | **Yes** | **Clear** | **9** | **High** |
| **22** | Larry Jones et al. (2017) | **Yes** | **Yes** | **Yes** | **Yes** | **Yes** | **Yes** | **Yes** | **Yes** | **Yes** | **Clear** | **9** | **High** |
| **23** | W.K.B.A. Owiredu et al. (2010) | **Yes** | **Yes** | **Yes** | **Yes** | **Yes** | **Yes** | **Yes** | **Yes** | **Yes** | **Not clear** | **8** | **Moderate** |
| **24** | Olivier Pancha Mbouemboue et al. (2016) | **Yes** | **Yes** | **Yes** | **Yes** | **Yes** | **No** | **Yes** | **Yes** | **Yes** | **Not clear** | **8** | **Moderate** |
| **25** | B Longo-Mbenza et al. (2008) | **Yes** | **Yes** | **Yes** | **Yes** | **Yes** | **Yes** | **Yes** | **Yes** | **Yes** | **Clear** | **9** | **High** |
| **26** | Edward Antwi, et al. (2016) | **Yes** | **Yes** | **Yes** | **Yes** | **Yes** | **Yes** | **Yes** | **Yes** | **Yes** | **Clear** | **9** | **High** |
| **27** | V. O. Osunkalu, et al (2019) | **Yes** | **Yes** | **Yes** | **Yes** | **Yes** | **Yes** | **Yes** | **Yes** | **Yes** | **Clear** | **9** | **High** |

CASP Key questions

1. Question 1- Did the study address a clearly stated objective?
2. Question 2- Was the study accurately measured to minimise bias?
3. Question 3- Was the target population specified ?
4. Question 4- Was the study use appropriate study method?
5. Question 5- How precise the tools used to measure the results?
6. Question 6- was the response rate adequate?
7. Question 7- Do the results of the study fit with other available evidence ?
8. Question 8- Can the results be applied to the local population?
9. Question 9- Does the paper answered objective clearly?
10. Question `10- What are the implications of this study for practice?
