# Supplementary material for: Factors associated with hypertensive disorders of pregnancy in sub-Saharan Africa: A systematic and meta-analysis
Source: PLoS One. 2020 Aug 19;15(8):e0237476. doi: 10.1371/journal.pone.0237476 (PMC7437911; doi:10.1371/journal.pone.0237476)
Supplement: S1 File — (DOCX) [file pone.0237476.s004.docx]

Funnel plots for associated factors of HDP in SSA

**Fig S1. Funnel plot on association between parity and HDP in subsequent pregnancies in SSA**

**Fig S2. Funnel plot on association between having previous HDP and HDP in subsequent pregnancies in SSA**

**Fig S3. Funnel plot on association between family history of HDP and HDP in subsequent pregnancies in SSA**

**Fig S4. Funnel plot on association between maternal education and HDP in subsequent pregnancies in SSA**

**Fig S5. Funnel plot on association between diabetes mellitus and HDP in subsequent pregnancies in SSA**
